# Anti-Inflammatory Effects of Heparin and Its Derivatives: A Systematic Review

**DOI:** 10.1155/2015/507151

**Published:** 2015-05-12

**Authors:** Sarah Mousavi, Mandana Moradi, Tina Khorshidahmad, Maryam Motamedi

**Affiliations:** ^1^Department of Clinical Pharmacy and Pharmacy Practice, Faculty of Pharmacy and Pharmaceutical Sciences, Isfahan University of Medical Sciences, Isfahan, Iran; ^2^Faculty of Pharmacy, Zabol University of Medical Sciences, Zabol, Iran; ^3^Faculty of Pharmacy, Tehran University of Medical Sciences, Tehran, Iran

## Abstract

*Background*. Heparin, used clinically as an anticoagulant, also has anti-inflammatory properties. The purpose of this systematic review was to provide a comprehensive review regarding the efficacy and safety of heparin and its derivatives as anti-inflammatory agents. *Methods*. We searched the following databases up to March 2012: Pub Med, Scopus, Web of Science, Ovid, Elsevier, and Google Scholar using combination of Mesh terms. Randomized Clinical Trials (RCTs) and trials with quasi-experimental design in clinical setting published in English were included. Quality assessments of RCTs were performed using Jadad score and Consolidated Standards of Reporting Trials (CONSORT) checklist. *Results*. A total of 280 relevant studies were reviewed and 57 studies met the inclusion criteria. Among them 48 studies were RCTs. About 65% of articles had score of 3 and higher according to Jadad score. Twelve studies had a quality score > 40% according to CONSORT items. Asthma (*n* = 7), inflammatory bowel disease (*n* = 5), cardiopulmonary bypass (*n* = 8), and cataract surgery (*n* = 6) were the most studied disease condition. Forty studies use unfractionated heparin (UFH) for intervention; the remaining studies use low molecular weight heparin (LMWH). *Conclusion*. Despite the conflicting results, heparin seems to be a safe and effective anti-inflammatory agent; although it is shown that heparin can decrease the level of inflammatory biomarkers and improves patient conditions, still more data from larger rigorously designed studies are needed to support use of heparin as an anti-inflammatory agent in clinical setting. However, because of the association between inflammation, atherogenesis, thrombogenesis, and cell proliferation, heparin and related compounds with pleiotropic effects may have greater therapeutic efficacy than compounds acting against a single target.

## 1. Introduction

Heparin is a highly sulfated glycosaminoglycan (GAG) that is found in the mast cells of most mammals. The endogenous GAGs are highly acidic and actively charged. Heparin is the most sulfated, and acidic GAGs enable it to bind to different component such as coagulating and fibrinolysing proteins, many growth factors, and immune response proteins such as cytokines and chemokines [1, 2]. Heparin is mostly known for its anticoagulant properties, so commercially form of heparin including unfractionated heparin (UFH) and low molecular weight heparin (LMWH) used currently in treatment and prevention of thrombotic events like deep vein thrombosis, pulmonary emboli, acute coronary syndromes, and ischemic cerebrovascular events as well as prevention of thrombosis in extracorporeal circuits and hemodialysis [3, 4]. Apart from its anticoagulant effects, there are several studies which have shown that heparin possesses various anti-inflammatory and immunomodulatory properties and the mechanisms of anti-inflammatory actions of heparin have been discussed recently [5, 6]. But the exact benefit and safety of heparin and its derivatives as anti-inflammatory agents in clinical setting have not definitely proved yet. Our objective was to systematically review and summarize the literature supporting anti-inflammatory role of heparin to provide evidence about the clinical effectiveness and safety of heparin in inflammatory conditions.

## 2. Methods

### 2.1. Search Strategy

A comprehensive literature search was conducted in PubMed, Scopus, Web of Science, Ovid, Elsevier, and Google Scholar from inception to March 2012 using the following Mesh keywords: (1) heparin, (2) UFH, (3) anticoagulants, (4) dalteparin, (5) enoxaparin, (6) nadroparin, (7) tinzaparin, (8) heparinoids, (9) inflammation, (10) inflammatory process, (11) anti-inflammation, (12) inflammation mediators, (13) inflammatory bowel disease, and (14) anti-inflammatory agents. All keywords from 1 to 8 were separately combined with each keyword from 9 to 14 in all databases. Articles were initially scanned based on titles and abstracts by two reviewers (Sarah Mousavi and Mandana Moradi) and related articles were retrieved in full and assessed for eligibility by two reviewers (Sarah Mousavi and Mandana Moradi). The reference list of each eligible study was checked to identify additional studies.

### 2.2. Inclusion Criteria

All Randomized Clinical Trials (RCTs) and studies with quasi-experimental design which evaluated efficacy, using inflammatory biomarkers levels, and safety (significant hemorrhage or thrombocytopenia) of anti-inflammatory effects of heparin and heparin-related derivatives (LMWH or other heparinoids) with an English abstract regardless of rout of administration (intravenous, subcutaneous, topical, or inhaler), age, gender, race, and ethnic origin of participants were included.

### 2.3. Exclusion Criteria

The following were excluded: studies based on animal models; preclinical and biological studies, letters, and editorials; report published as meeting abstract only; where insufficient data were reported to allow inclusion.

### 2.4. Data Extraction and Quality Assessment

Data from each eligible study were extracted individually and compared by two authors (Sarah Mousavi and Mandana Moradi) using standard form that included study design, setting, sample size, duration and follow up, dosing regimen, intervention type, and outcomes. Disagreements were resolved through discussion; if necessary they consulted a third person. A narrative synthesis was conducted.

Quality assessment of clinical trials included in the analysis were performed utilizing the Jadad score, a previously validated instrument that assesses trials based on appropriate randomization, blinding, and description of study withdrawals or dropouts [7]. The description of this score is as follows: (1) whether it is randomized (yes = 1 point, no = 0); (2) whether randomization was described appropriately (yes = 1 point, no = 0); (3) whether it is double-blind (yes = 1 point, no = 0); (4) whether the double-blinding was described appropriately (yes = 1 point, no = 0); (5) whether withdrawals and dropouts were described (yes = 1 point, no = 0). The quality score ranges from 0 to 5 points; a low-quality report score is ≤2; and a high-quality report score is at least 3.

The Consolidated Standards of Reporting Trials (CONSORT) checklist was also used for randomized trials as it is strongly endorsed by prominent journals and leading editorial organizations [8]. Total possible score for CONSORT checklist was considered as 74: two points for adequate description, one point for inadequate description, and zero for no description.

### 2.5. Statistical Methods

To summarize and extract data, the database was designed by Microsoft office Access 2007 (Microsoft Corporation, Redmond, WA). A narrative synthesis was conducted and data were extracted into tables and summarized.

## 3. Results

**Figure 1:**
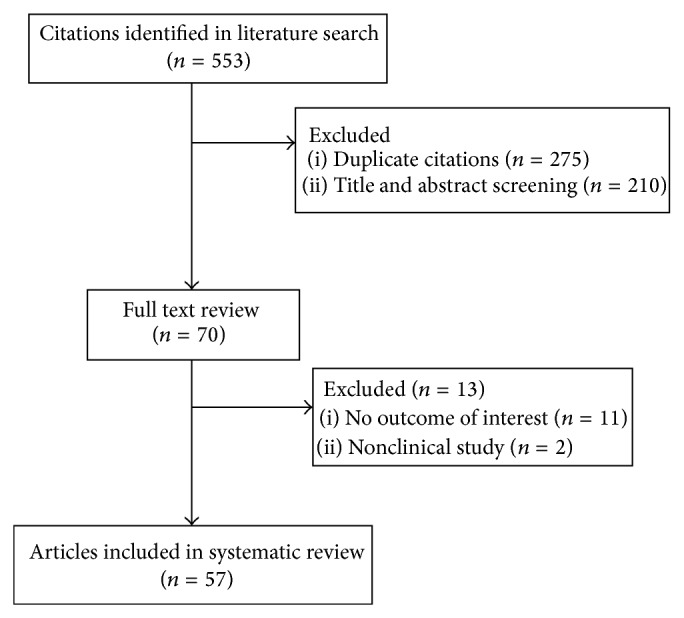
Flow diagram of literature search process.

Following Initial screening of mentioned databases total of 553 citations (275 duplicates) were extracted but only 70 of them were potentially eligible for investigation of our objectives (according to our proposed inclusion criteria) based on titles and abstracts. The full text screening excluded other 13 citations and the remaining 57 papers were considered relevant for data extraction and following analysis. The flow chart of studies' selection processes is as shown in Figure 1.

### 3.1. Study and Patient Characteristic

Sample sizes ranged from 8 to 555 patients in 57 studies that met the criteria to be included. Research designs mostly were randomized controlled trial (*n* = 48) and the remaining (*n* = 9) had quasi-experimental designs using pre-post studies (*n* = 6).

Tables 1 and 2 [9–62] list the characteristics of the included studies. The most studied clinical conditions was cardiopulmonary bypass (*n* = 12), followed by Asthma (*n* = 8), inflammatory bowel disease (IBD) (*n* = 5), acute coronary syndrome (ACS) (*n* = 8), and ophthalmological disease (*n* = 8). Other less common studied conditions were as follows: burn, cystic fibrosis, allergic rhinitis, superficial thrombophlebitis, and hemodialysis. Unfractionated heparin (UFH) was used in forty studies as subcutaneous, intravenous, inhaler, or heparin-coated circuits while others used enoxaparin (*n* = 3), dalteparin (*n* = 4), nadroparin (*n* = 4), and tinzaparin (*n* = 1).

Table 3 provides information on the adequately reported items according to Jadad score and CONSORT items. Among these 32 papers, 11 (35.4%) scored 2 and the remaining studies scored 3 or higher according to Jadad score and just three studies fulfill the criteria of Jadad score. Calculated quality scores according to CONSORT checklist range from 21% to 70% in our object studies with twelve studies scoring greater than 40%. The following characteristics were not exactly reported in more than half of the trials: identification as a randomized trial in the title, information about the setting and location of studies, determination of sample size, allocation and implementation of randomization, participant flow, recruitment and follow-up, subgroup analysis, limitations of study, harms, registration number, access to full trial protocol, and funding source.

## 4. Discussion

We discuss evidence from clinical studies supporting an anti-inflammatory role for heparin and heparin-related derivatives.

### 4.1. Asthma

Asthma is a chronic inflammatory disorder of airways characterized by bronchial hyperresponsiveness resulting in episodic bronchospasm. Several studies in 1960s described subjective improvement of symptoms in asthmatic patients using intravenous heparin for the first time [39, 63]. Inhaled heparin or its derivatives have been shown to possess antiasthmatic properties in various clinical models: allergen induced [38, 64], exercise [9], adenosine [6], and distilled water challenge models [65]. Seven randomized controlled crossover trials studied anti-inflammatory effects of heparin in exercise-induced or allergen-induced asthma having sample size range from 8 to 25 in 5 trials.

Heparin inhalation reduced bronchial hyperreactivity in a single-blind randomized crossover trial (*n* = 12) by Ahmed et al. [9]. Heparin inhalation (1000 *μ*/kg) prevents exercise-induced asthma (*P* < 0.05) without prevention of histamine-induced bronchoconstriction. Stelmach et al. [66] provoke challenge tests with histamine or leukotriene D4 before and after inhalation of heparin; showed that heparin (5000 Iu) decreases histamine and leukotriene-induced bronchial hyperreactivity compared to placebo significantly (*P*: 0.043 and 0.005) but changes in Forced Expiratory Volume (FEV 1) were not significant (*P*: 0.064). The inhibitory effects of inhaled heparin in the airways in the absence of bronchodilation might be related to suppressive action on mast cell degranulation. A randomized, double-blind study in 10 subjects with asthma showed that heparin inhalation attenuates airway response to adenosine 5′-monophosphate (AMP) but not to methacholine (*P* < 0.01) suggesting the theory that heparin acts more likely in association to modulation of mediator release compared to a direct effect on smooth muscle [67].

Our results showed that inhaled enoxaparin was used just in a pre-post study of 24 asthmatic patients, measuring inflammatory biomarkers, and showed a reduction in eosinophil (*P*: 0.006) and lymphocytes of bronchoalveolar lavage without any significant change in IL-5 or Eosinophil Cationic Protein (ECP) concentrations [12]. Therefore, it seems that enoxaparin could be a valuable add on treatment in asthma like UFH. Duong et al. [11] evaluated IVX-0142 nebulizer, a heparin-derived hypersulfated disaccharide, in asthma and showed nonsignificant decrease in early and late asthmatic response.

Performed studies did not show any adverse events or harms with heparin or related compounds except increase in the plasma partial thromboplastin time reported by Ahmed et al. [9].

All in one, considering the results of these studies, we can conclude that heparin and its derivatives could have anti-inflammatory effects and could be considered along with other treatments in asthma.

### 4.2. Cardiopulmonary Bypass

Contact and interaction of blood with foreign surfaces during cardiopulmonary bypass (CPB) cause systemic inflammatory response syndrome (SIRS) through activation of several humoral cascades including cytokines such as IL-6, IL-8, and Tumor Necrosis Factor-*α* (TNF-*α*) [68]. The inflammatory response can be attenuated by promoting the biocompatibility of the CPB circuit. Use of heparin-treated surfaces in CPB circuits has been shown to decrease activation of leukocytes and the complement cascades [5]. As a result, need to inotropic support, postoperative time of mechanical ventilation, and rate of acute lung injury decrease and patients duration of hospital stay shortens, which reflects the positive effect of heparin in CPB circuits [69, 70]. Twelve studies whose endpoints were the effects of heparin-bonded circuits on inflammatory markers were included in our analysis and in the majority of these trials [27, 29, 32–35, 71]; heparin-coated circuit significantly decreased the level of cytokines such as IL-6, IL-8, TNF-*α*, complement complex, neutrophils, and elastase compared to non-heparin-coated circuit.

Defraigne et al. [28] randomized 200 patients in 4 groups; heparin-coated circuit with or without aprotinin administration and uncoated circuit with or without aprotinin administration. They measured IL-6, IL-8, TNF-*α*, myeloperoxidase (MPO), and elastase level and concluded that cytokine release and neutrophil activation were not attenuated by heparin-coated circuit or by the administration of aprotinin. Misawa et al. [30] evaluated cytokines level under normothermic CPB, in a small observational controlled study (*n* = 19, 9 in control group). Levels of IL-6, IL-8, and ICAM-1 (indicator of endothelial damage) were not different between study and control group. However, as mentioned before, most studies indicate the favorable effect of heparin-coated circuit on inflammatory responses in CPB.

Paparella et al. [72] compared two doses of heparin and 300 Iu/kg and 600 Iu/kg in 40 patients undergoing CPB in an RCT and showed that IL-6 and TNF-*α* plasma levels were in association to heparin dose. It seems that lower heparin dose had small influence on proinflammatory cytokines release; however, higher doses make a better regulatory effect on coagulation system.

The side effects of heparin-coated circuits were not reported. In these studies, just a number of included studies reported a decrease in platelet levels in both groups (coated and noncoated circuit). No major events including hemorrhage were reported.

### 4.3. Inflammatory Bowel Diseases (IBD)

Hypercoagulable state may be an important contributing factor in the pathogenesis of IBD, especially ulcerative colitis (UC) [73]. A number of studies evaluate the effects of heparin administration on UC but the results obtained are controversial [17, 19]. Bloom et al. [74] did not find any favorable effect of LMWH, tinzaparin, over placebo in the treatment of active UC in a double-blind randomized, placebo controlled, multicenter trial (*n* = 100) evaluating mean change in colitis activity as the primary endpoint. Ang et al. [17] compared heparin to hydrocortisone plus prednisolone in 20 patients (UC, *n* = 17, Crohn's colitis, *n* = 3) in open-label randomized crossover trial which measured endpoints; clinical disease activity, stool frequency and *α*_1_ acid glycoprotein, and endoscopic and histopathological grading indicate the efficacy and safety of heparin compared to corticosteroids (*P* > 0.05); in contrast the study by Panes et al. [19] did not show the efficacy of heparin in UC compared to methylprednisolone.

Zezos et al. [20] compared enoxaparin with standard treatment (aminosalicylate + corticosteroids) in 34 patients with active UC. The inflammatory biomarkers including C-Reactive Protein (CRP), Erythrocyte Sedimentation Rate (ESR), and fibrinogen did not show any difference in study group compared to control and both groups showed similar rate of disease improvement (*P* > 0.05); furthermore, coagulation factors did not change from one to another. Authors concluded that enoxaparin is a safe adjuvant but has no additive benefit over standard treatment of UC.

Generally the studies show conflicting results. The heparin and LMWHs showed efficacy in regard of disease activity and also well tolerated but inflammatory markers did not change significantly. Therefore the improvement in disease activity might be the result of heparin's anticoagulant effects.

### 4.4. Acute Coronary Syndrome (ACS)

Inflammation has a key role in the pathogenesis of coronary artery plaque destabilization and rupture leading to acute coronary syndromes (ACS) [75]. Leukocyte activation, monocyte, and neutrophil infiltration result in local and systemic inflammatory responses [76]. Heparin and LMWHs are commonly used in ACS to prevent clot formation; they also seem to have desirable effects on inflammatory markers level based on sparse data.

We found 8 studies about heparin and LMWHs in CADs evaluating anti-inflammatory effects as their endpoints. Oldgren et al. [56] found that dalteparin administration in patients with unstable CAD (*n* = 555) did not affect IL-6, C-Reactive Protein (CRP), and fibrinogen levels, although it reduced coagulation activity and mortality rate in long term, so it is concluded that these effects are not related to its anti-inflammatory properties. Walters et al. [58] compared high dose of tirofiban/enoxaparin (*n* = 30) with tirofiban/heparin (*n* = 30) in an open-label randomized controlled trial in high risk percutaneous intervention. They found that combination of high dose of tirofiban with enoxaparin significantly attenuate inflammatory process (decreased levels of CRP, and von Willebrand factor) compared to tirofiban and heparin.

The ARMADA study [54] evaluated anti-inflammatory effects of heparin and enoxaparin in Non-ST-Elevated Myocardial Infarction (Non-STEMI) and reported that inflammatory markers (CRP and von Willebrand factor) are affected more by LMWH compared to UFH. However because of the small sample size of the study (*n* = 68) it did not acquire sufficient statistical power to prove any effects on defined outcomes. Nasiripour et al. [55] found that both enoxaparin and heparin reduce inflammatory markers in STEMI patients at the same level; however, this study had the same limitations of sample size (*n* = 34) and power too.

In summary considering heparins as the main stay treatment of ACS, its effects are more pronounced as anticoagulating than as an anti-inflammatory agent in this pathological condition.

### 4.5. Cataract Surgery

Postoperative inflammation is observed in cataract surgery especially in children. Newer techniques as lensectomy and phacoemulsification cause less complication but still pose potential risks [77, 78]. It has been suggested that a heparin surface-modified intraocular lens (IOL) or augmenting the irrigating solution with heparin during cataract surgery may reduce the incidence of postoperative inflammation. Borgioli et al. [22] confirm this hypothesis that heparin surface-modified IOL will reduce the inflammatory response compared to conventional IOL at least in short term period (3 months) in a large (524 patients) double-blind, multicenter trial. A similar study by Colin et al. [23] (*n* = 58) confirms their results; however, the power of Borgioli et al. study was higher. Heparinized lenses showed also more anti-inflammatory effects during long term follow-up (1 year). Heparinized irrigating solution was used during cataract surgery in two different studies and inflammation decrease observed in the early postoperative period of both studies (Kohnen et al. [26] and Özkurt et al. [24]). Therefore, it seems that heparin in both forms have anti-inflammatory effects in cataract surgery.

Vasavada et al. [25] used enoxaparin irrigation solution in 20 children undergoing bilateral cataract surgery but they did not find any beneficial effect in early postoperative inflammation. This study acquired the highest quality in the study quality assessment process, based on Jadad score and CONSORT items (52/74, 70.2%). It is worth noticing that the majority of items mentioned in the CONSORT checklist were adequately reported and covered in this paper.

Potential mechanisms of anti-inflammatory effects of heparin have been discussed completely in a review by Young [6]. Binding of heparin to different mediators involved in the immune system response (cytokines and chemokines), acute phase proteins, and complement complex proteins may contribute to the anti-inflammatory activity of heparin. Neutralizing of cytokines at the inflammation site is another possible mechanism. In most of the studies level of cytokines such as IL-6, IL-8, TNF-*α*, and CRP was decreased after heparin administration, which can confirm this mechanism; also heparin and LMWHs inhibit adhesion of leukocytes and neutrophils to endothelial cells by binding to p-selectin, and consequently prevent release of oxygen radicals and proteolytic enzymes. Other possible mechanisms are as follows: inhibition of nuclear factor *κ*B (NF-*κ*B) and induction of apoptosis by modulation of activity of TNF-*α* and NF-*κ*B. In a double-blind placebo-controlled trial (*n* = 62) in patients with stable coronary artery disease, following administration of 1 mg/kg subcutaneous enoxaparin, plasma levels of myeloperoxidase (MPO) increased significantly (*P* < 0.001) and subsequently endothelial function improved (*r* = 0.67, *P* < 0.001) through MPO binding to endothelium and depleting vascular nitric oxide [57]. This study confirms another possible mechanism of heparins anti-inflammatory effects.

## 5. Conclusion

As we discussed heparins potential effects as anti-inflammatory agents, supported by several clinical trials in various setting. Heparin and its related derivatives have been shown to benefit patients with asthma and patients undergoing cardiopulmonary bypass and cataract surgery. In other inflammatory diseases, such as IBD (ulcerative colitis), the studies are heterogeneous and incongruent. Most studies did not report any unwanted event with heparins when they used them as anti-inflammatory agents whether through systemic or through local (as inhaler or irrigation or heparin-coated circuit) administration. However, because the majority of these trials did not pose optimal quality scores, we cannot draw a definite conclusion on the efficacy of heparin and its derivatives as anti-inflammatory agents.

The present review included studies which measured inflammatory markers as their endpoints and in most of them these markers were decreased though not significantly. To come to a definite conclusion further double-blind, randomized, placebo-controlled clinical trials with a larger sample size are needed. However, because the inflammation, atherogenesis, thrombogenesis, and cell proliferation are associated with each other, the pleiotropic effects of heparin and related compounds may have greater therapeutic effect than compounds that are directed against a single target.

## Figures and Tables

**Table 1:** Summary and findings of common studied diseases.

Clinical setting	Heparin preparation	Mode of administration	Comparator	Number of patients	Clinical outcome	Laboratory outcome	Study design
Exercise-induced Asthma [9]	UFH	Inhaler	Cromolyn sodium or placebo	12	Significantly reduction of exercise-induced asthma	Heparin had no effect on histamine-induced bronchoconstriction	Single-blind, randomized, crossover clinical trial

Asthma [10]	UFH	Inhaler	Placebo	8	Significant reduction of late asthmatic response after allergen administration (*P*: 0.005)	—	Randomized, double-blind, crossover clinical trial

Atopic asthma [11]	Heparin (IVX-0142)	Nebulizer	Placebo	19	No significant decrease in early (*P*: 0.06) and late (*P*: 0.24) asthmatic response	—	Randomized single-blind, placebo-controlled, crossover trial

Asthma [12]	LMWH	Nebulizer	—	24	Effective as an add-on therapy to standard treatment	Reduction in eosinophil (*P*: 0.0006) and lymphocyte (*P*: 0.049) in bronchoalveolar lavage. No changes in IL-5 or ECP concentrations in serum	Quasi-experimental (pretest-posttest design)

Allergic asthma [13]	UFH	Inhaler	Placebo	25	Heparin inhalation significantly reduced bronchial hyperreactivity (*P* < 0.05)	—	Randomized, double-blind, placebo-controlled, crossover trial

Asthma [14]	UFH	Inhaler	—	12	Transient (time-dependent) inhibitory role in allergic reactions	Increased the methacholine PC20 value (*P*: 0.05) but did not prevent an increase in Raw and/or a decrease in SGAW	Randomized, double-blind, placebo-controlled, crossover trial

Asthma (children) [15]	UFH	Inhaler	Placebo	14	Single dose of heparin significantly (*P*: 0.005) reduced bronchial hyperreactivity	Provocation test used leukotriene D4	Randomized, double-blind, placebo-controlled, crossover trial

Asthma [16]	UFH	Inhaler	Placebo	23	Significant reduction of bronchial hyperreactivity to histamine and leukotriene	—	Randomized, double-blind, placebo controlled, crossover trial

IBD [17]	UFH	IV/SC	Hydrocortisone + prednisolone	20 (12 in control group)	Clinical activity index, stool frequency, and endoscopic and histopathological grading were similar in both treatment groups	CRP and *α*1 acid glycoprotein did not change	Open label randomized, crossover clinical trial

IBD [18]	UFH	SC	—	17	Histology improved significantly in ulcerative colitis patients (UFH is effective in ulcerative colitis but not Crohn disease)	CRP (*P*: 0.0119) and ESR (*P*: 0.0096) significantly reduced in ulcerative colitis but not Crohn disease	Quasi-experimental (pretest-posttest design)

IBD [19]	UFH	IV	Methyl prednisolone	25 (13 in control group)	No effect of heparin, also increased bleeding	No change in CRP	Randomized, double-blind, parallel-group trial

IBD [20]	Enoxaparin + standard treatment	SC	Aminosalicylate + corticosteroid	34 (18 in control group)	Significant improvement in disease severity in both groups (*P*: 0.001)	No difference ESR, CRP and fibrinogen and coagulation	Randomized controlled trial

IBD [21]	Nadroparin	SC	—	25	Endoscopic and histological sign of inflammation significantly improved	—	Quasi-experimental (Non-randomized clinical trial)

Cataract surgery [22]	UFH	Intraocular lens (IOL)	Polymethylmethacrylate	524	—	Heparin surface modification reduced the cellular deposit compared to control group	Randomized, double-blind, parallel group clinical trial

Cataract surgery [23]	UFH	Intraocular lens (IOL)	Polymethylmethacrylate	58 (31 in control group)	Postoperative inflammation decreased significantly in heparin group (*P*: 0.02)	Giant cell and cell deposit decreased significantly (*P* < 0.05)	Randomized, double-blind, clinical trial

Cataract surgery (pediatric) [24]	UFH	Irrigation	Balanced salt solution	33 (19 in control group)	Heparin irrigation reduced number of postoperative inflammatory related complication	Anterior chamber reaction including fibrin formation was lower in heparin group	Randomized prospective double-blind trial

Cataract surgery (pediatrics) [25]	Enoxaparin	Irrigation	No treatment	40 (20 in each group)	Increase of flare and cell deposit after surgery (1 and 3 months) (*P*: 0.99)	Increase in large cell deposits	Randomized, double-blind, controlled trial

Cataract surgery [26]	UFH	Irrigation	Regular irrigation solution	72	Significant reduction of inflammation in the early (days 1–3) postoperative period (*P* < 0.01)	—	Randomized controlled trial

Cardiopulmonary bypass (pediatric) [27]	Heparin-coated circuit (*n* = 11)	—	Non-heparin-coated circuit (*n* = 10)	21	Decrease of systemic inflammatory response with the use of heparin-bonded oxygenators	Significantly decreased levels of IL-6, IL-8, terminal complement complex, neutrophils, and elastase in heparin coated circuit	Randomized controlled trial

Cardiopulmonary bypass [28]	Heparin-coated circuit ± aprotinin	—	Uncoated circuit ± aprotinin	200 (4 groups)	Aprotinin and heparin had no effect on cytokine release	TNF-*α*, IL-6, and IL-8 and myeloperoxidase did not change	Randomized, double-blind, clinical trial

Cardiopulmonary bypass [29]	UFH	—	Uncoated circuit	51 (26 in each group)	Decreased pulmonary vascular resistance index and pulmonary shunt fraction, and increased PaO2/FIO2 ratio	Lower levels of phospholipase A2 and complement activation (*P*: 0.001)	Randomized, double-blind, clinical trial

Cardiopulmonary bypass [30]	Heparin-coated circuit	—	Non-heparin-coated circuit	16 (9 in control group)	—	No significant difference between groups regarding: granulocyte elastase IL-6, IL-8	Quasi-experimental (pretest-posttest design)

Cardiopulmonary bypass [31]	Heparin concentration-based system	—	Activated clotting time-based management	200 (100 in control group)	No effect on postoperative blood loss	Significant reduction of neutrophil activation and fibrinolysis and thrombin generation (*P* < 0.05)	Randomized controlled trial

Cardiopulmonary bypass (pediatric) [32]	Heparin-coated circuit	—	Non-heparin-coated circuit	19 (10 in control group)	Improvement of the biocompatibility of CPB during heart surgery	Levels of complement factor C3a (*P* < 0.001) and IL-6 (*P*: 0.005) significantly reduced in heparin-coated circuit	Randomized controlled trial

Cardiopulmonary bypass (pediatric) [33]	Heparin-coated circuit	—	Non-heparin-coated circuit	34 (12 in control group)	No differences in duration of intubation, intensive care unit or hospital stay, or postoperative blood loss	IL-6, IL-8, and TNF-*α* were significantly lower in heparin group (*P* < 0.01, *P* < 0.01, and *P* < 0.05, resp.)	Randomized controlled trial

Cardiopulmonary bypass [34]	Heparin-coated circuit (heparin + aprotinin)	—	Non-heparin-coated circuit (heparin + aprotinin)	30 (15 in each group)	No significant differences between the two groups in terms of bleeding and transfusional requirements, the time spent on a ventilator, or in duration of stay in the intensive care unit (ICU)	Levels of IL-6, CRP, and neutrophil count did not change by heparin-coated circuit. Monocyte count increased in heparin-coated circuit	Randomized controlled trial

Coronary artery bypass grafting (CABG) [35]	Heparin-coated circuit	—	Non-heparin-coated circuit	18 (9 in each group)	—	Reduction of levels of IL-8 and TNF-*α* and increase of neutrophil elastase	Randomized controlled trial

CRP: C-Reactive Protein, CPB: Cardio Pulmonary Bypass, ECP: Eosinophil Cationic Protein, ESR: Erythrocyte Sedimentation, ICU: Intensive Care Unit, IL: interleukin, IV: intravenous, SC: subcutaneous, SGAW: Specific Airway Conductance, TNF: Tumor Necrosis Factor, and UFH: unfractionated heparin.

**Table 2:** Summary and findings of other studied diseases.

Clinical setting	Heparin preparation	Mode of administration	Comparator	Number of patients	Clinical outcome	Laboratory outcome	Study design
Pancreatitis after ERCP [36]	UFH	SC	Saline solution	105 (54 in control group)	Rate of postoperative pancreatitis was not significant between both groups	—	Randomized placebo-controlled clinical trial

Acute coronary syndrome (ACS) [37]	UFH	SC	Enoxaparin	201	—	No significant difference between CD4 ligand and PAI-1 in both groups	Open label, randomized, clinical trial

Skin or pulmonary allergy [38]	UFH	IV/nebulizer	Normal saline/placebo	25	Significant inhibition of mast cell-mediated allergic inflammation (*P*: 0.04)	—	Double-blind, placebo-controlled, crossover clinical trial

COPD [39]	UFH	IV	—	37 (18 in control group)	Significant improvement in bronchospasm and bronchial secretions (58% response rate)	—	Randomized placebo-controlled clinical trial

COPD [40]	Nadroparin	SC	Conventional treatment	66 (33 in each group)	Decrease of duration of mechanical ventilation and length of hospital and ICU stay (*P* < 0.01)	Significant decrease in levels of CRP, IL-6, and fibrinogen	Randomized controlled trial

Ischemic stroke [41]	UFH	IV	Aspirin	167 (97 in control group)	Early onset initiation of heparin might improve recovery after stroke	Rise of sVCAM-1 at 48 h was significantly lower in patients treated with UFH (*P* < 0.01)	Quasi-experimental (controlled observational study)

Ligneous conjunctivitis [42]	UFH	Topical	Alpha chymotrypsin or steroid	17 (12 in control group)	Intensive and early use of topical heparin may improve therapy results in disease	—	Quasi-experimental (nonrandomized Clinical trial)

Endotoxemia (induced by lipopolysaccharide in healthy subjects) [43]	UFH	IV	LMWH or placebo	30 (10 in each group)	—	No effect on TNF-*α*, IL-6 and 8, CRP, and E-selectin	Randomized, double-blinded, placebo-controlled parallel group trial

Mechanical ventilation [44]	UFH	Nebulizer	Normal saline (placebo)	50 (25 in each group)	Fewer days on mechanical ventilation, better Pao2/Fio2 ratio	—	Double-blind, randomized, placebo-controlled trial

Percutaneous coronary intervention [45]	Bivalirudin	IV	UFH + eptifibatide	63 (29 in control group)	—	Increase in IL-6 and CRP after 1 day. Decrease in CRP in bivalirudin group after 30 days (*P*: 0.002)	Randomized controlled trial

Cystic fibrosis (adults) [46]	UFH	Inhaler	—	12 (6 in control group)	Spirometry parameters did not change	IL-6 reduced after treatment	Quasi-experimental (pretest-posttest design)

Cystic fibrosis (adults) [47]	UFH	Inhaler	Placebo	14	No effect on FEV1	No effect on sputum inflammatory markers	Randomized, double-blind, placebo-controlled crossover trial

Hemodialysis patients [48]	UFH	IV/SC	LMWH and no drug	33	LMWH decreased oxidative stress and inflammation whereas heparin increased them	CRP, TNF-*α*, superoxide dismutase, MDA increased in heparin group but comparable to LMWH group	Quasi-experimental (pretest-posttest design)

Stable angina [49]	Heparin + Aspirin (*n* = 15)	—	Argatroban + Aspirin (*n* = 12) 27		No difference in inflammatory response after angioplasty	Fibrinogen decreased significantly in argatroban group. No difference in von Willberand factor between both groups. PAI-1 increased in argatroban group	Randomized controlled trial

Phacoemulsification [50]	Heparin	Coated lenses	Polymethylmethacrylate lenses	367	Heparin coated lenses reduced significantly inflammation early postoperation (*P*: 0.05)	—	Randomized, double-blind, multicenter, parallel group trial

Allergic rhinitis [51]	UFH	Intranasal	—	10	—	Reduction of eosinophil cationic protein in the nasal wash	Quasi-experimental (pretest-posttest design)

Phacomorphic glaucoma [52]	Dalteparin	Irrigation	Balanced salt solution	46 (23 in each group)	Significant decrease of postoperative inflammation in dalteparin group	—	Randomized, double-blind, clinical trial

Burn [53]	Dalteparin	SC	No treatment	24	—	Decrease of nitric oxide synthetase activity significantly	Quasi-experimental (nonrandomized clinical trial)

Unstable coronary artery disease [54]	Enoxaparin (*n* = 46) Dalteparin (*n* = 48)	SC	UFH (*n* = 47)	68	Von Willberand factor may have prognostic value, but other biological variables did not predict outcome	CRP, fibrinogen, Von Willberand factor increased over first 2 days despite medical treatment. Enoxaparin (13%) and dalteparin (19%) reduced release of Von Willberand factor	Open label, randomized, clinical trial

ST-Elevated Myocardial Infarction (STEMI) [55]	Enoxaparin	SC	UFH	34 (17 in each group)	Both heparin and enoxaparin show anti-inflammatory effects in STEMI patients	Serum Amyloid A (*P*: 0.02), CRP (*P*: 0.02), and ferritin (*P*: 0.01) reduced in heparin group. IL-6 (*P*: 0.002), SAA (*P*: 0.009), CRP (*P*: 0.01) were significantly decreased in enoxaparin group. The overall difference between groups was not significant	Open label, randomized, clinical trial

Coronary artery disease [56]	Dalteparin	SC	Placebo	555 (285 in control group)	Dalteparin reduced coagulation and so Myocardial Infarction but has not inflammatory activity	No effects on IL-6, C-reactive protein and fibrinogen	Randomized, double-blind, parallel-group, multicentre trial

Stable coronary artery disease [57]	Enoxaparin	SC	Sodium chloride	62 (31 in each group)	By mobilizing vessel bound MPO, enoxaparin improves endothelial function	Significant increase of MPO levels	Randomized, double-blind, placebo-controlled trial

Acute coronary syndrome and PCI [58]	Tirofiban (high dose) + enoxaparin		Tirofiban (high dose) + UFH	60 (30 in each group)	The combination of tirofiban (high dose) + enoxaparin reduced inflammation after PCI	Von willberand, CRP, D-dimer, and prothrombin fragment were significantly lower in enoxaparin group than UFH	Open label randomized controlled trial

Superficial venous thrombophlebitis [59]	Dalteparin	SC	Ibuprofen	72 (37 in dalteparin group)	Significant reduction of pain form baseline to day 14 of follow-up. No difference on thrombosis progression after 3 months	—	Randomized, double-blind, controlled trial

Superficial venous thrombosis [60]	Nadroparin	SC	Naproxen	117 (39 in control group)	Nadroparin reduced symptom and signs of thrombosed superficial vein better than naproxen (*P*: 0.007)	—	Randomized, open label clinical trial

Superficial venous thrombosis [61]	Nadroparin	SC	Nadroparin + acemetacin	50	Significant symptom improvement in both groups (*P*: 0.001). The combination group was better	—	Randomized controlled trial

Peritoneal dialysis patients [62]	Tinzaparin	Intraperitoneal	Isotonic saline	21	Reduction of local and systemic inflammation in peritoneal dialysis patients	Reduced levels of CRP (*P*: 0.032) and fibrinogen (*P*: 0.042) and IL-6 (*P*: 0.007) in dialysate.	Randomized, double-blind, placebo-controlled crossover trial

COPD: Chronic Obstructive Pulmonary Disease, CRP: C-Reactive Protein, CPB: Cardio Pulmonary Bypass, ECP: Eosinophil Cationic Protein, ERCP: Endoscopic Retrograde Cholangiopancreatography, ESR: Erythrocyte Sedimentation, ICU: Intensive Care Unit, IL: interleukin, IV: intravenous, LMWH: low molecular weight heparin, MDA: malondialdehyde, PAI: Plasminogen Activator Inhibitor, SC: subcutaneous, sVCAM: Soluble Vascular Cell Adhesion Molecule, TNF: Tumor Necrosis Factor, and UFH: unfractionated heparin.

**Table 3:** Summary of numbers and percentages of adequately reported items in each trial according to CONSORT checklist and Jadad score.

Trials	Jadad score	Adequately reported items (*n*/*N*)	Percentage (%)
Abdollahi et al. [52]	4	29/74	39.2%
Ahmed et al. [9]	3	20/74	27%
Ang et al. [17]	2	24/74	32.4%
Ashraf et al. [27]	2	22/74	29.7%
Becker et al. [37]	3	28/74	37.8%
de Vroege et al. [29]	3	26/74	35.1%
Defraigne et al. [28]	4	28/74	37.8%
Derhaschnig et al. [43]	3	32/74	43.2%
Dixon et al. [44]	4	50/74	67.5%
Duong et al. [11]	3	30/74	40.5%
Gu et al. [71]	2	16/74	21.6%
Jerzynska et al. [13]	3	20/74	27%
Keating et al. [45]	2	21/74	28.4%
Koster et al. [31]	2	26/74	35.1%
Montalescot et al. [54]	3	35/74	47.3%
Nasiripour et al. [55]	2	34/74	46%
Oldgren et al. [56]	3	27/74	36.5%
Olsson et al. [32]	2	25/74	33.8%
van Ophoven et al. [79]	2	27/74	36.5%
Ozawa et al. [33]	2	27/74	36.5%
Özkurt et al. [24]	3	18/74	24.3%
Paparella et al. [72]	2	22/74	29.7%
Polosa et al. [67]	3	23/74	31.1%
Rathbun et al. [59]	5	44/74	59.5%
Rudolph et al. [57]	3	31/74	41.2%
Serisier et al. [47]	5	45/74	60.8%
Stelmach et al. [16]	3	31/74	41.2%
Suzuki et al. [49]	2	23/74	31%
Vancheri et al. [51]	4	22/74	29.7%
Vasavada et al. [25]	5	52/74	70.2%
Walters et al. [58]	3	32/74	43.2%
Zezos et al. [20]	3	37/74	50%
